# HIV and suicide risk across adolescence and young adulthood: an examination of socio‐demographic, contextual and psychosocial risk factors for attempted suicide in a longitudinal cohort of ageing adolescents affected by HIV living in the New York City Area

**DOI:** 10.1002/jia2.25984

**Published:** 2022-09-29

**Authors:** Philip Kreniske, Corey Morrison, Bailey Holmes Spencer, Alina Levine, Lucy Liotta, Prudence W. Fisher, Nadia Nguyen, Reuben N. Robbins, Curtis Dolezal, Luke Kluisza, Andrew Wiznia, Elaine J. Abrams, Claude A. Mellins

**Affiliations:** ^1^ HIV Center for Clinical and Behavioral Studies New York State Psychiatric Institute and Columbia University New York City New York USA; ^2^ Department of Population and Family Health Mailman School of Public Health Columbia University New York City New York USA; ^3^ Mental Health Data Science Research Foundation for Mental Hygiene New York City New York USA; ^4^ Child and Adolescent Psychiatry New York State Psychiatric Institute and Columbia University New York City New York USA; ^5^ The Aaron Diamond AIDS Research Center New York City New York USA; ^6^ Jacobi Medical Center Albert Einstein College of Medicine Bronx New York USA; ^7^ ICAP at Columbia University Mailman School of Public Health and Vagelos College of Physicians & Surgeons Columbia University New York City New York USA

**Keywords:** HIV, young adults, adolescents, HIV stigma, suicide, cohort studies

## Abstract

**Introduction:**

As children become adolescents and young adults (AYA), their risk for attempting suicide increases dramatically, with chronic health conditions an important risk factor. This study examined correlates of suicidality across development in AYA living with perinatally acquired HIV (AYALPHIV) and those perinatally HIV‐exposed but uninfected (AYAPHEU).

**Methods:**

Data come from an ongoing longitudinal New York City‐based study (*N* = 339) with AYALPHIV and AYAPHEU interviewed every 12–18 months from 2003 to 2019 (mean enrolment age = 12 years; current mean age = 27 years). The Diagnostic Interview Schedule for Children (adolescent or young adult version) assessed psychiatric disorders and first‐reported suicide attempt. Generalized estimating equations were used to examine associations between first‐reported suicide attempt and socio‐demographic, contextual and psychosocial correlates measured concurrently across six timepoints.

**Results:**

At enrolment, 51% of participants were female, 72% heterosexual, 60% Black and 50% Latinx. Attempted suicide was significantly higher among AYALPHIV (27%, CI 21–33%) compared to AYAPHEU (16%, CI 10–22%), with an OR of 1.74 (CI 1.04–2.92) in a model adjusting for age. For AYALPHIV, anxiety (OR 2.66, CI 1.46–4.85), mood (OR 3.62, CI 1.49–8.81) and behaviour disorders (OR 5.05, CI 2.15–11.87) and past‐year arrest (OR 3.05, CI 1.26–7.4), negative life events (OR 1.27, CI 1.11–1.46), city stress (OR 2.28, CI 1.46–3.57), pregnancy (OR 2.28, CI 1.08–4.81) and HIV stigma (OR 2.46, CI 1.27–4.75) were associated with increased odds of attempted suicide, while identifying as heterosexual (OR 0.27, CI 0.14–0.52), higher personal (OR 0.45, CI 0.26–0.80) and family self‐concept (OR 0.36, CI 0.22–0.57) were protective. Interactions by HIV status and age were found: substance use was more strongly associated with attempted suicide among AYAPHEU than AYALPHIV, while negative life events and higher religiosity were more strongly associated with increased odds of attempted suicide among AYA ≥ 19 versus ≤ 18 years.

**Conclusions:**

AYALPHIV compared to AYAPHEU faced unique risks for attempted suicide as they age into adulthood, with the highest risk among AYALPHIV experiencing HIV stigma or pregnancy and the highest risk among AYAPHEU with substance use. Assessing for suicide risk and correlates with attention to ageing can inform preventive interventions tailored to meet AYALPHIV and AYAPHEU needs.

## 1 INTRODUCTION

Risk of attempting suicide increases dramatically as children become adolescents and young adults (AYA) (10–30 years) [1, 2, 3, 4, 5]. During this time, symptoms of mental illness and substance use often emerge [6], increasing the risk for suicidal thoughts and behaviours [7, 8, 9, 10, 11, 12]. Social (e.g. racial, gender‐based discrimination) [3, 13–18] and contextual (e.g. neighbourhood violence) [19, 20, 21] factors may also contribute to increased suicide risk among specific sub‐groups of AYA, such as sexual and racial minorities. Youth with chronic illnesses have shown higher suicidality in some studies [22], including AYA affected by HIV [23], yet, no studies to our knowledge have investigated proximal risk factors for suicidality among AYA affected by HIV as they age across this critical developmental period [24]. Given the staggering numbers globally of young people growing up with HIV or affected by maternal HIV [25, 26], understanding suicidality in this population is critical to informing much‐needed evidence‐based prevention interventions [27, 28], particularly because the history of attempted suicide is a strong predictor of later death by suicide [29, 30, 31].

During adolescence, identity development incorporates sexual, racial and ethnic identity, in the context of lived environments [32, 33, 34, 35, 36, 37]. This process includes internalizing values and conceptualizing how identity may impact social experiences and life opportunities [35, 38]. Sexual and racial/ethnic minorities are at increased risk for suicidality because of identity‐based discrimination, which has the strongest impact during adolescence and young adulthood [3, 13–18, 39]. For people living with HIV (PLWHIV), establishing an identity can be especially complex as they grapple with a stigmatized, chronic, transmittable infection [40, 41].

A recent systematic review and meta‐analysis found that PLWHIV, median age 39 years, had a 100‐fold higher risk of dying by suicide than people not living with HIV, with an even higher risk for people living with AIDS [42]. While risk factors for suicide in the general population of AYA are well documented [43, 44, 45], few studies have examined suicidality among AYA living with perinatally acquired HIV (AYALPHIV) [24] and those that have showed mixed results [46, 47, 48, 49, 50].

To further our understanding of predictors of suicide attempts, we examined longitudinal data (adolescence through young adulthood) from the Child and Adolescent Self‐Awareness and Health study (CASAH). CASAH was informed by Social Action Theory (SAT) [51], which considers individual, social and contextual determinants of behavioural health outcomes. The objective of the current analysis was to identify proximal risk and protective socio‐demographic, contextual and psychosocial factors associated with suicide attempts in AYALPHIV and AYA who were perinatally HIV‐exposed but uninfected (AYAPHEU).

## 2 METHODS

### 2.1 Study population

CASAH is an ongoing longitudinal cohort study, now in its 19th year, that includes AYALPHIV (*n* = 207) and AYAPHEU (*n* = 133) recruited from four New York City (NYC) medical centres between 2003 and 2008 when participants were ages 9–16 years (mean = 12). One AYAPHEU was excluded from the current analyses because they seroconverted during the study. Detailed methods have been described elsewhere [52, 53]. In brief, inclusion criteria were perinatal HIV exposure; cognitive capacity to complete interviews; English or Spanish‐speaking; and caregiver with the legal capacity to sign consent for adolescent participation (AYA < 18 provided assent; those ≥ 18 years informed consent). Providers referred eligible clinical patients to the study; 93% of all participants approached enrolled. The current analyses include data collected until 2019 from participants’ psychosocial battery at six timepoints, with participants aged 21–32 years (mean = 27) at the most recent visit. Interviews were administered by trained research assistants and participants were compensated for time and travel expenses. The study was approved by the New York State Psychiatric Institute Institutional Review Board.

### 2.2 Study measures

We selected risk and protective factors informed by SAT and identified by studies on adolescent suicide, HIV and mental health [7, 9–12, 22–24, 29, 47, 49, 54–67]. Unless otherwise indicated, variables for the following age‐appropriate measures that had been used in other studies of PLWHIV and racially and ethnically diverse US populations were assessed at all timepoints. When applicable, we provide Chronbach's alpha as a measure of reliability (Table 1) [68, 69]. We then examined data on potential correlates of the first reported suicide attempt collected from the same timepoint as the report for those who endorsed a suicide attempt.

**Table 1:** Description of measures and timepoints collected in the longitudinal cohort of adolescents and young adults affected by HIV living in the New York City Area

Measure	Scale name	Measure age group	Cronbach's α	Enrol	FU1	FU2	FU3	FU4	FU5
First attempted suicide	The Diagnostic Interview Schedule for Children (adolescent or young adult version) (DISC) [70]	Administered by age (< 18 years received the child version, > 18 years received a slightly modified young adult version)	NA	X	X	X	X	X	X
Age, race/ethnicity, HIV status, sexual orientation, past year pregnancy	N/A	All ages	NA	X	X	X	X	X	X
Recent arrest or incarceration	Monitoring the Future [78]	All ages	NA			X	X	X	X
Young adult negative life events	Children's Life Events Inventory [72]; Life Events Questionnaire [75]	Developed with children ages 8–15 years [72], but items are appropriate for young adults and have been used in other studies of youth living with HIV [75]	NA^a^	X	X	X	X		X
City stress	City Stress Inventory (CSI) [77]	Appropriate for those with an approximate eight‐grade reading level [77]	NA^a^	X	X	X	X	X	X
Religiosity	Adapted from Monitoring the Future [78]	All ages	0.77	X	X	X	X	X	X
Self‐concept	Tennessee Self‐Concept Scale [81]	All ages	Personal: 0.72 Family: 0.78 Social: 0.53	X	X	X	X		
Psychiatric disorder	Diagnostic Interview Schedule for Children (DISC) [70]	Administered by age (< 18 years received the child version, > 18 years received a slightly modified young adult version)	NA	X	X	X	X	X	X
HIV stigma	Social Impact Scale [82], HIV Stigma Scale [147] (FU5)	Appropriate for all ages. Before FU5, Social Impact Scale was only asked to PHIV who's caregivers disclosed youth knew about PHIV status.	0.84	X	X	X	X	X	X

Abbreviation: FU, follow up.

^a^ Chronbach's alpha computed at enrolment. The city stress index and life events scale both consist of items that are combined into an index following a formative measurement framework, in which the items are the cause of the construct rather than the effect, and therefore, do not follow a reflective measurement framework. Therefore, Chronbach's alpha is not appropriate for these measures [68,69].

#### 2.2.1 Suicidality

Suicidality was assessed at each visit using one item from the well‐validated Diagnostic Interview Schedule for Children (DISC) [70] “Have you ever in your whole life tried to kill yourself or make a suicide attempt? (yes/no).” All participants who reported any suicidality, including intentions were evaluated for active suicidal ideation; no participants required being taken to the emergency room, although mental health treatment referrals were routinely made.

#### 2.2.2 Socio‐demographic factors

Socio‐demographic variables at enrolment included sex (female/male), age, sexual orientation (heterosexual/not), perinatal HIV status (positive/negative), race and ethnicity, and history of pregnancy in self or partner.

#### 2.2.3 Contextual factors

##### 2.2.3.1 Negative life events (measured at enrolment‐FU3)

This measure consists of 18 negative events (e.g. parents divorced and death of family member) typically considered adverse childhood experiences [71], developed by one of the authors (CAM) and providers at a family‐based HIV mental health programme for the target population [72, 73], with higher scores indicating more adverse life events. A total score was created and has been used in multiple papers [74, 75, 76].

##### 2.2.3.2 City Stress Inventory

The 16‐item City Stress Inventory assesses perceived neighbourhood stress, specifically urban stressors (e.g. witnessing drug deals and gang violence) appropriate for AYA with an eight grade reading level [77]. AYA reported how frequently they experienced these neighbourhood stressors in the past year using a 4‐point scale [0 = never; 4 = often]. Higher scores indicated a higher level of neighbourhood stress.

##### 2.2.3.3 Recent arrest or incarceration (measured FU2‐5)

A dichotomous variable was created based on AYA self‐report of an arrest, spent time in jail or incarceration in the past year, with items from Monitoring the Future, a well‐validated measure of adolescent risk behaviour [78].

#### 2.2.4 Psychosocial factors

##### 2.2.4.1 Psychiatric disorders

We assessed psychiatric disorders with the DISC, which has well‐validated adolescent (< 18 years) and young adult (> 18 years) versions [79]. Symptoms experienced in the past year for the most common psychiatric diagnoses were assessed, adhering to the Diagnostic and Statistical Manual of Mental Disorders [80] diagnostic criteria. The following broad diagnostic categories were examined: mood disorders, anxiety disorders, behaviour disorders and substance use disorders.

##### 2.2.4.2 Religiosity

Participants reported their religious affiliation, and, using 4‐point Likert scales, past year frequency of participation in religious activities, belief in a higher power and the importance of religion or spirituality in their life appropriate for AYA [78]. A total religiosity score was created ranging from 0 to 18, with higher scores indicating higher religiosity (Cronbach's α = 0.77).

##### 2.2.4.3 Self‐concept (enrolment‐FU3)

Self‐concept was measured using the Tennessee Self‐Concept Scale:2 (TSCS:2), appropriate for AYA [81]. The TSCS:2 is composed of self‐descriptive items, each answered on a 5‐point Likert Scale (0 = not at all; 5 = very much); higher scores reflect better self‐concept. We used three sub‐scales: personal self‐concept (e.g. “I'm a cheerful person” Cronbach's α = 0.72), family self‐concept (e.g. “I am a member of a happy family” Cronbach's α = 0.78) and social self‐concept (e.g. “I am a friendly person” Cronbach's α = 0.53).

##### 2.2.4.4 HIV stigma

Among AYALPHIV, HIV stigma was assessed with the Social Impact Scale appropriate for AYA [82]. Using 18 items from a 4‐point Likert‐type scale, participants reported how much they agreed with statements concerning social rejection, isolation and internalized shame related to HIV (e.g. “I feel I need to keep my HIV a secret”), with higher scores indicating higher levels of HIV‐related stigma (Cronbach's α = 0.84).

### 2.3 Statistical analysis

Data were set in a long format with participants contributing multiple observations over time. Descriptive statistics were calculated and AYALPHIV and AYAPHEU participants were compared on enrolment socio‐demographic and psychosocial variables. *T*‐tests and chi‐squared tests were used for continuous and categorical variables, respectively. Additionally, we plotted the cumulative incidence (1‐Kaplan–Meier) of the time to first suicide attempt with age as the x‐axis.

Given the rare outcome of suicide attempt and the use of repeated measures over time, we used logistic regression with generalized estimating equations (GEE) to individually estimate odds ratios (OR) and 95% confidence intervals (CI) for the association between each socio‐demographic, contextual and psychosocial factor and having the first report of attempted suicide (binary outcome), adjusting for age at each survey round [83, 84]. Repeated observations were modelled using an independent working correlation matrix, but empirical standard errors were reported, which are robust to the misspecification of the correlation structure. We examined these associations in the overall sample which included both AYALPHIV and AYAPHEU, and among AYALPHIV and AYAPHEU sub‐groups. Overall sample models were additionally adjusted for HIV status (PHIV or PHEU), and HIV stigma was only examined among AYALPHIV. Similar to the way, censoring is handled in grouped proportional hazards model [85, 86, 87], for participants who ever reported a lifetime suicide attempt, observations were included in the model up until (and including) the observation in which a lifetime suicide attempt was first reported. We explored potential interactions by developmental stage by including an interaction term for ages 9–18 and 19 and older, and by PHIV‐status by including a PHIV‐status interaction term in analyses with the overall sample. Analyses were completed using SAS version 9.4 [88].

## 3 RESULTS

At enrolment, 51% of participants were female (*n* = 172), 72% heterosexual (*n* = 227), 60% Black (*n* = 203) and 50% Latinx (*n* = 170); 11% were Black and Latinx (*n* = 36) and two participants were neither Black nor Latinx) with no significant differences between AYALPHIV and AYAPHEU (Table 2). Participants were followed for a mean of 8.5 years (minimum = 0 years [only enrolment], maximum = 15.9 years). The number of participants reporting attempted suicide increased from 15 (8 females and 7 males) at enrolment to 76 (37 females and 39 males) at the most recent visit. Participants were on average 19 years old (standard deviation = 4.4) at first reported suicide attempt.

**Table 2:** Description of the overall cohort and AYA living with perinatally acquired HIV (AYALPHIV) and AYA who were perinatally HIV‐exposed but uninfected (AYAPHEU) subgroups at enrolment

	Overall sample (*N* = 339)^b^		AYALPHIV (*n* = 206)		AYAPHEU (*n* = 133)		Diff between groups
Variable	*n*	Mean (SD) or %^b^	*n*	Mean (SD) or %^b^	*n*	Mean (SD) or %^b^	*p*‐value
Attempted suicide^a^	76	22% (18%, 27%)	55	27% (21%, 33%)	21	16% (10%, 22%)	**0.02**
*Socio‐demographic factors*							
Sex							0.91
Male	167	49%	102	50%	65	49%	
Female	172	51%	104	50%	68	51%	
Identifies as straight/heterosexual	227	72%	130	69%	97	77%	0.11
Black/African‐American	203	60%	131	63.59%	72	54.14%	0.08
Latinx	170	50%	99	48.06%	71	53.38%	0.34
Black and Latinx							
Age	339	12.58 (2.25)	206	12.70 (2.16)	133	12.38 (2.37)	0.19

Abbreviations: AYALPHIV, adolescents and young adults living with perinatally acquired HIV; AYAPHEU, adolescents and young adults perinatally HIV‐exposed but uninfected.

Bold indicates *p* < 0.05.

^a^ Attempted suicide considers all survey rounds.

^b^ *N*s may not sum to total due to missing data. Percentages are of those with non‐missing data. Percentages may not sum to 100 due to rounding.

**Figure 1:**
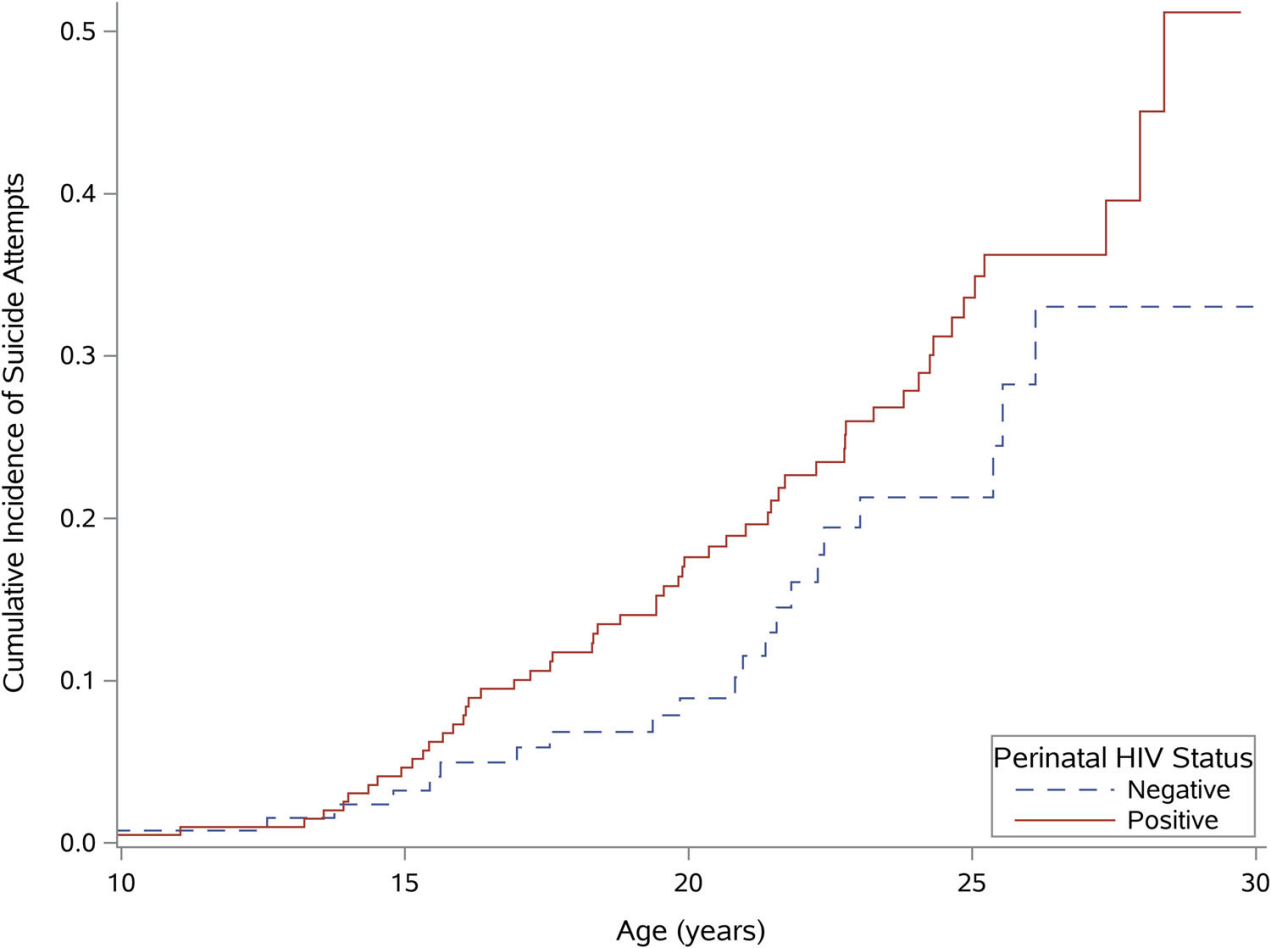
Kaplan–Meier cumulative incidence estimates of first suicide attempt among AYA living with perinatally acquired HIV (AYALPHIV) and AYA who were perinatally HIV‐exposed but uninfected (AYAPHEU).

Attempted suicide prevalence was significantly higher among AYALPHIV (27%, 95% CI 21–33%) compared to AYAPHEU (16%, 95% CI 10–22%, *p* = 0.02) (Table 2). In the age‐adjusted logistic marginal model accounting for participants’ repeated observations, AYALPHIV had 1.74 times higher odds than AYAPHEU of ever attempting suicide (95% CI 1.04–2.92) (Table 3). Figure 1 shows the Kaplan–Meier cumulative incidence estimates of first suicide attempt among AYALPHIV and AYAPHEU in our sample. Based on these estimates, by the time a child reached 17 (the median age of all observations), the cumulative incidence of suicide attempt was 6% for AYAPHEU and 10% for AYALPHIV.

**Table 3:** Associations between first report of attempted suicide and socio‐demographic, structural and psychosocial factors for the overall cohort and within AYA living with perinatally acquired HIV (AYALPHIV) and AYA who were perinatally HIV‐exposed but uninfected (AYAPHEU) subgroups

	Odds ratio (95% CI)^a^		
Variable	Overall	AYALPHIV	AYAPHEU
HIV status			
Positive	**1.74 (1.04, 2.92)**		
Negative (ref)			
Socio‐demographic factors			
Sex			
Male	0.8 (0.5, 1.28)	0.74 (0.42, 1.3)	1.00 (0.4, 2.45)
Female (ref)			
Identifies as straight/heterosexual			
Yes	**0.30 (0.17, 0.51)**	**0.27 (0.14, 0.52)**	0.37 (0.13, 1.04)
No (ref)			
Race			
Black/African‐American	1.12 (0.68, 1.82)	1.28 (0.7, 2.32)	0.79 (0.33, 1.89)
Not Black/African‐American (ref)			
Ethnicity			
Latinx	1.38 (0.86, 2.22)	1.21 (0.69, 2.12)	2.02 (0.8, 5.07)
Not Latinx (ref)			
Ever experienced pregnancy			
Yes	1.84 (0.98, 3.45)	**2.28 (1.08, 4.81)**	1.13 (0.35, 3.67)
No (ref)			
*Structural factors*			
Arrested/spent time in jail past year			
Yes	**2.56 (1.16, 5.67)**	**3.05 (1.26, 7.4)**	1.20 (0.14, 10.13)
No (ref)			
Negative life events	**1.23 (1.09, 1.39)**	**1.27 (1.11, 1.46)**	1.08 (0.85, 1.36)
City Stress Inventory	**2.07 (1.4, 3.06)**	**2.28 (1.46, 3.57)**	1.62 (0.74, 3.53)
*Psychosocial factors*			
Religiosity	1.23 (0.93, 1.64)	1.14 (0.82, 1.58)	1.52 (0.85, 2.72)
Personal self‐concept	**0.51 (0.32, 0.83)**	**0.45 (0.26, 0.8)**	0.68 (0.28, 1.64)
Family self‐concept	**0.39 (0.26, 0.59)**	**0.36 (0.22, 0.57)**	0.51 (0.24, 1.09)
Social self‐concept	1.10 (0.71, 1.72)	1.19 (0.68, 2.07)	0.87 (0.45, 1.68)
DISC anxiety disorder			
Yes	**2.88 (1.76, 4.7)**	**2.66 (1.46, 4.85)**	**3.43 (1.46, 8.07)**
No (ref)			
DISC mood disorder			
Yes	**4.39 (2.29, 8.41)**	**3.62 (1.49, 8.81)**	**5.88 (2.33, 14.88)**
No (ref)			
DISC behaviour disorder			
Yes	**4.70 (2.39, 9.23)**	**5.05 (2.15, 11.87)**	**4.06 (1.35, 12.17)**
No (ref)			
DISC substance disorder			
Yes	**2.60 (1.49, 4.73)** ^b^	1.83 (0.89, 3.78)^b^	**5.83 (2.23, 15.29)** ^b^
No (ref)			
*HIV‐specific factors*			
HIV stigma		**2.46 (1.27, 4.75)**	

Abbreviations: AYALPHIV, adolescents and young adults living with perinatally acquired HIV; AYAPHEU, adolescents and young adults perinatally HIV‐exposed but uninfected; DISC, Diagnostic Interview Schedule for Children [70].

^a^ OR greater than 1 indicates a greater likelihood of attempted suicide. OR adjusted for age at each survey round and overall models were adjusted for PHIV status.

^b^ Interaction by PHIV status was significant (*p* = 0.041).

Bold indicates *p* < 0.05.

### 3.1 Socio‐demographic factors

In the age‐adjusted and HIV status‐adjusted logistic marginal models accounting for repeated observations, for the overall and AYALPHIV populations, those who identified as heterosexual had lower odds of attempted suicide compared to those who did not identify as heterosexual (Overall: OR = 0.3, 95% CI, 0.17–0.51; AYALPHIV: OR = 0.27, 95% CI 0.14–0.52). Frequencies of specific non‐heterosexual identities were too small to examine separately.

### 3.2 Contextual factors

For the overall sample and AYALPHIV group, contextual factors significantly associated with increased odds of attempted suicide were city stress (Overall: OR = 2.07, 95% CI 1.4–3.06; AYALPHIV: OR = 2.28, 95% CI 1.46–3.57; negative life events (Overall: OR = 1.23, 95% CI 1.09–1.39; AYALPHIV: OR = 1.27, 95% CI 1.11–1.46); and having been arrested or spent time in jail in the past year (Overall: OR = 2.56, 95% CI 1.16–5.67; AYALPHIV: OR = 3.05, 95% CI 1.26–7.40).

### 3.3 Psychosocial factors

Among the overall and AYALPHIV populations, higher personal self‐concept (Overall: OR = 0.51, 95% CI 0.32–0.83; AYALPHIV: OR = 0.45, 95% CI 0.26–0.80) and family self‐concept (Overall: OR = 0.39, 95% CI 0.26–0.59; AYALPHIV: OR = 0.36, 95% CI 0.22–0.57) were protective against attempted suicide. Overall, psychosocial factors associated with increased odds of attempted suicide included having any anxiety disorder (Overall: OR = 2.88, 95% CI 1.76–4.7); AYALPHIV: OR = 2.66, 95% CI 1.46–4.85; AYAPHEU: OR = 3.43, 95% CI 1.46–8.07); any mood disorder (Overall: OR = 4.39, 95% CI 2.29–8.41); AYALPHIV: OR = 3.62, 95% CI 1.49–8.81; AYAPHEU: OR = 5.88, 95% CI 2.33–14.88); or any behaviour disorder (Overall: OR = 4.70, 95% CI 2.39–9.23; AYALPHIV: OR = 5.05, 95% CI 2.15–11.87; AYAPHEU: OR = 4.06, 95% CI 1.35–12.17). For the overall and AYAPHEU populations, substance use disorder was associated with higher odds of attempted suicide and we found evidence of interaction by PHIV‐status (*p* = 0.04) with substance use as a greater risk factor for attempted suicide in the AYAPHEU group (Overall: OR = 2.60, 95% CI 1.49–4.73; AYAPHEU: OR = 5.83, 95% CI 2.23–15.29). No significant association was found in the AYALPHIV group.

Among AYALPHIV only, pregnancy and HIV stigma were associated with increased odds of attempted suicide. A unit increase in HIV stigma was associated with 2.46 times higher odds of attempted suicide (95% CI: 1.27–4.75). AYALPHIV who experienced pregnancy had 2.28 times higher odds of attempted suicide compared to those who never experienced pregnancy (95% CI: 1.08–4.81). A gender‐by‐pregnancy interaction term was tested, as it was hypothesized that associations between pregnancy and attempted suicide would be higher for females; the interaction term was not significant (*p* = 0.90).

### 3.4 Interaction by PHIV status and developmental stage

Among older AYA (≥ 19 years), each negative life event increased the odds of attempted suicide by a factor of 1.42 (95% CI: 1.15–1.76) with no significant association among younger AYA (OR = 1.13, 95% CI: 0.97–1.33) (interaction *p*‐value = 0.09). Among older AYA, each unit increase in religiosity score increased the odds of attempted suicide by a factor of 1.60 (95% CI: 1.11–2.31), with no significant association among younger AYA (OR = 0.83, 95% CI: 0.54–1.28) (interaction *p*‐value = 0.03).

## 4 DISCUSSION

Adolescence and young adulthood is a high‐risk period for attempting suicide [1, 2, 3, 4], with AYALPHIV a particularly vulnerable group [49, 50]. The current findings extend the field, not only by providing additional evidence that AYALPHIV are at greater risk for suicide attempts across adolescence and into young adulthood than AYAPHEU peers [23, 49, 50], but also by showing proximal risk and protective factors for both groups as they age through this developmental period. For AYALPHIV, stigma and pregnancy were associated with attempted suicide and for AYAPHEU, substance use was a risk factor. Although our data suggest shared findings with other populations of AYA [67, 89–91], differences were identified that suggest strategies for suicide prevention tailored for AYA affected by HIV.

The association between pregnancy (self or partner) and attempted suicide among AYALPHIV in our study is an important finding for preventive interventions. Prior studies suggest younger age among HIV‐negative pregnant females is a risk factor for suicidality [91, 92, 93, 94, 95]. Adult women living with HIV have reported concerns regarding vertical HIV transmission, providing for a child while living with HIV, or fears of mortality [96, 97, 98], which may also be relevant to AYALPHIV of all genders. Future studies should examine the timing of pregnancy in this population and mental health effects, including suicidality.

Similar to studies with the general population, we found that non‐heterosexual identity was significantly associated with attempted suicide in both AYALPHIV and AYAPHEU [3, 18]. Extensive research has demonstrated associations between sexual identity discrimination and poor mental health outcomes, with AYA sexual minorities being at the greatest risk for suicidality [3, 17, 18]. Further research could inform interventions by exploring how anti‐LGBT discrimination might interact with feelings about growing up with HIV and negatively impact AYA social and emotional development.

We identified associations between attempted suicide and contextual factors, including recent arrests, city stress and negative life events in the full cohort, with negative life events having a stronger association with attempted suicide among those who were 19 or older. Interventions for older AYA may be able to stem the harm from exposure to negative life events [99, 100], yet to date, no programmes exist specifically tailored to AYA affected by HIV [27, 28]. Further, these contextual factors may be more salient for Black and Latinx AYA given that they are more likely [53] than White AYA to live in low‐income neighbourhoods where negative life events (e.g. family member death, neighbourhood violence and recent arrest) have been associated with attempted suicide [19, 20, 101] and are more common as compared to higher‐income neighbourhoods [21, 102–106]. Neighbourhood discrepancies by race and ethnicity are likely the result of systemic racism affecting housing and policing policies, the detrimental impact of which has been documented [64, 107–111]. Unfortunately, we could not examine racial differences as our participants were predominantly Black and Latinx. Additional research among ethnic and racial minorities affected by HIV and suicidality is needed.

Prior study of suicidality among AYA affected by HIV has found mixed results. Three cross‐sectional studies (from England, Thailand and South Africa) found no differences in suicide risk between AYALPHIV versus those without HIV [46, 47, 48]. Conversely, our current and prior work in the United States [50] and a study in Rwanda [49] both found an increased risk of suicidality among AYALPHIV compared to AYA without HIV. Differences in the HIV epidemic and affected populations across nations [112, 113, 114], as well as cross‐cultural differences, such as the impact of systemic discrimination on people with intersectional identities, including racial and ethnic minorities living with HIV in the United States, could explain the previously noted mixed findings internationally regarding HIV‐status and adolescent suicidality [115, 116, 117].

As found in our study, stressful events can also lead to other negative developmental outcomes [21, 102–105], including mental illness, a known correlate of attempted suicide among AYA [7–12, 118]. Self‐concept, related to mental health, is an important psychosocial factor that changes during adolescence and young adulthood. Although positive self‐concept appears protective against suicidality in other populations, it has not been previously examined over time among AYALPHIV and AYAPHEU. We examined self‐concept across different domains [81]. Consistent with international and US studies that suggest family processes influence suicidality among PLWHIV [58, 119], and among adolescents not affected by HIV [12, 66, 67, 120], higher levels of family self‐concept were protective against attempted suicide among the overall sample and AYALPHIV. In addition, personal self‐concept among the overall sample and AYALPHIV was protective, aligning with prior findings among HIV‐negative youth [121, 122].

There were also psychosocial risk factors unique to AYAPHEU and AYALPHIV. Substance use was a risk factor for attempted suicide only among AYAPHEU, supporting prior studies that highlight AYAPHEU as a vulnerable group with unique risks that may require tailored interventions [41, 123, 124]. For AYALPHIV, HIV stigma was a risk factor for attempted suicide. Interestingly, as in our prior work among this cohort, we did not find significant associations between HIV stigma and suicide attempts [50]. Early data from our cohort suggested few participants told others about their HIV which might have limited exposure to stigma. This changed as young people aged, perhaps because of increased sexual behaviour, potentially resulting in more opportunities for stigmatizing experiences [37].

Notably, higher religiosity was associated with higher odds of attempted suicide among AYA over 18 years of age, yet in previous CASAH analyses, we found that religiosity was protective against attempted suicide in earlier adolescence. The changing direction of this association warrants further study and suggests that as our cohort ages, religiosity may shift from a neutral or protective factor to a risk factor for attempted suicide. This changing dynamic between religiosity and attempted suicide as our cohort ages may reflect changes in sexuality, stigmatizing experiences or family dynamics, and corresponds with prior mixed findings from studies of suicide and religiosity in the general population [125, 126].

The longitudinal design of the current study was a strength, allowing us to examine our aims as our cohort transitioned into adolescence and young adulthood. Our use of standardized and validated measures, including the DISC, to assess mental health and attempted suicide was another strength [70, 127, 128].

There were also limitations. Larger samples, including participants in other countries, would be helpful in detecting more nuanced group differences and moderators of our findings, particularly larger samples of AYAPHEU. Additionally, our study did not include an HIV‐unexposed cohort, thus, limiting the ability to examine the impact of perinatal HIV exposure or familial HIV on suicidality. Over a lifetime, approximately 4.6% of US adults attempt suicide [129], although according to a systematic review, the proportion may be as high as 3.1–8.8% among adolescents in the United States [130], with an international study including 90 countries noting a range of 10–15% [131]. Both AYAPHEU and AYALPHIV appear to have an elevated risk of attempted suicide compared to HIV‐unaffected populations. Furthermore, as has been noted in previous work, AYALPHIV have had better access to mental health services through their ongoing HIV care systems than AYAPHEU [53, 132], potentially minimizing group differences. In addition, our findings may not be generalizable to AYALPHIV and AYAPHEU living outside NYC. However, NYC is one of the epicentres of the United States. HIV epidemic among women and children, with 22% of US paediatric HIV cases from NYC; moreover, the demographics of CASAH participants are similar to those reported in national studies of AYAPHIV and AYAPHEU [53, 133–135]. Further, our results align with prior studies in sub‐Saharan Africa that suggest the importance of mental health in understanding suicidality among youth affected by HIV [24, 47, 49, 60].

## 5 CONCLUSIONS

Adolescence and young adulthood is a critical period when the risk for attempted suicide rises precipitously [1, 2, 3, 4]. We found that as our cohort aged, several socio‐demographic, contextual and psychosocial factors placed AYA at increased risk for suicidality and that only a higher self‐concept was protective. More negative life events and religiosity appeared to increase the risk of attempted suicide for participants over 18 years. Specific risks for attempted suicide for AYALPHIV were HIV stigma and pregnancy, with substance use a risk for attempted suicide among AYAPHEU. There is an urgent need to meet the needs of AYA affected by HIV and highlighting these risks for healthcare providers could be a needed first step towards preventive interventions [27, 28].

In the context of the COVID‐19 pandemic, we suspect the risk of suicidality for this cohort and other AYA affected by HIV may increase. Even before COVID, the multifaceted impact of HIV, poverty and racism contributed to high rates of behavioural health problems, health disparities and among AYALPHIV, low rates of viral suppression [136, 137, 138, 139], with few available evidence‐based interventions to support behavioural health or HIV treatment adherence [27, 28]. The disproportionate burden of COVID‐19 on Black and Latinx communities in the United States, and global COVID‐related barriers to treatment among PLWHIV, may exacerbate behavioural health problems and limit access to services for chronic health conditions, such as HIV and psychiatric disorders, thus potentially contributing to increased risk for suicidality among AYA affected by HIV [111, 140–146].

## COMPETING INTERESTS

The authors declare that they have no competing interests.

## AUTHORS’ CONTRIBUTIONS

CM, LL, LK, CAM and CD performed the research. CAM, EJA, AW and CD designed the research study. CD managed the data. PWF contributed essential diagnostic tools and expertise. AL analysed the data. AW and EJA assisted in recruiting participants. PK, CM and BHS wrote a first draft of the manuscript. RNR, NN, CAM, EJA, AW and PWF wrote key sections of the manuscript and provided critical comments and insights. All authors contributed to interpreting the final results, reviewing and revising, and all authors have approved the final manuscript.

## FUNDING

This research was supported by the National Institute of Mental Health (R01MH06913 PI Mellins and P30MH43520 PI Remien). Kreniske was also supported by K01MH122319 (PI Kreniske) and a New York State Office of Mental Health Policy Scholar Award. The funders had no role in study design, data collection and analysis, decision to publish or preparation of the manuscript.

## Data Availability

Due to privacy and ethical concerns the data cannot be made available because of the sensitivity of the HIV data, and the relatively small sample and ease of identifying people if a few demographics are known.
